# How Much Time Should Be Waited and What Are the Main Findings to Evaluate the Hepatocellular Carcinoma Response to Regorafenib? A Real-Life Experience

**DOI:** 10.1155/2021/6219896

**Published:** 2021-01-10

**Authors:** Gustavo Hideki Kawanami, Leopoldo Katsuda, Thiara Barcelos Rocha, Fabio da Silva Yamashiro, Leonardo Pelafsky, Xingshun Qi, Fernando Gomes Romeiro

**Affiliations:** ^1^Fundação para o Desenvolvimento Médico e Hospitalar (FAMESP) Hospital Estadual de Bauru, São Paulo, Brazil; ^2^Imagem Diagnósticos Médicos, Bauru, São Paulo, Brazil; ^3^Gastroenterology Division, Department of Internal Medicine, Faculdade de Medicina de Botucatu, Universidade Estadual Paulista (UNESP), São Paulo, Brazil; ^4^Surgery Department, Faculdade de Medicina de Botucatu, Universidade Estadual Paulista (UNESP), São Paulo, Brazil; ^5^Department of Gastroenterology, General Hospital of Northern Theater Command (Formerly General Hospital of Shenyang Military Area), Shenyang 110840, China

## Abstract

**Background:**

Hepatocellular carcinoma is a relevant cause of mortality worldwide, mainly among patients who have a prior liver disease. In spite of clear recommendations regarding surveillance and screening methods, most patients are still diagnosed only when they are no longer candidates to curative treatment modalities, while others do not achieve the goals of such treatments, thus increasing the need of anticancer drugs. Moreover, when cirrhotic patients begin to receive these drugs, many types of adverse events are seen as a reason to withdrawal, even when there are findings suggesting a good response to the treatment. *Case Summary*. This case report is about a cirrhotic patient who received many types of treatment, from surgery and chemoembolization during early stages to first- and second-line systemic therapy when the disease turned to be advanced. Since he had no signs of liver dysfunction and suffered tumor progression during sorafenib treatment, regorafenib was initiated. The main findings that make this case important are the adverse events after taking this second-line agent, which would certainly be considered unacceptable and would lead to the drug withdrawal. The reasons why regorafenib was maintained are explained based on clinical and imaging findings, showing how this decision led to an excellent response.

**Conclusions:**

The knowledge of the main adverse events described in the pilot clinical trials can avoid unnecessary withdrawal of regorafenib. In addition, some clinical and imaging findings can be deemed as predictors of good response to tyrosine kinase inhibitors.

## 1. Introduction

Sorafenib remains the first-line therapy for patients with advanced hepatocellular carcinoma (HCC) and unfortunately the incidence of the disease is still growing, so the need of second-line treatments is a serious issue. Ten years after sorafenib approval, the best drug to be used after its failure is another multikinase inhibitor with very similar chemical structure. Thus, tyrosine kinase inhibitors (TKI) are the main class of systemic agents in HCC treatment [1]. Moreover, according to the current guidelines, regorafenib is the only recommended drug after progression while on sorafenib usage if the patient has preserved liver function (Child-Pugh A) and good performance status and was tolerant to sorafenib [2]. Additionally, in a retrospective cohort, the best candidates to receive a second-line treatment were those with normal plasmatic levels of albumin with no (or minimal) decrease 4 weeks after taking sorafenib [3].

However, the questions about how much time and what are the best signals of tumoral response during regorafenib treatment are still under debate. This paper reports a case in which the initial treatment findings could lead many doctors to stop the treatment, moving the patient classification forward to terminal disease. The details about these findings and the reasons why the treatment was maintained will be depicted and deeply discussed.

## 2. Materials and Methods

**Figure 1:**
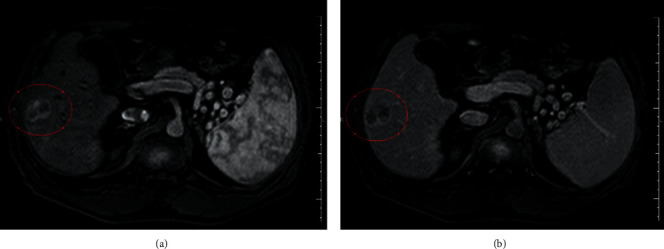
Magnetic resonance imaging performed on March 31, 2017, showing the initial liver tumor that measured 3.0 × 2.5 cm in liver segment VI with arterial enhancement and a marked washout in the portal phase (the tumor is underscored by the red line). (a) Arterial phase. (b) Portal phase.

The patient is a 64 year-old Caucasian man with liver cirrhosis caused by hepatitis C who had received antiviral treatment in 2016, achieving sustained virological response. A screening liver ultrasonography in February 2017 showed a suspected hepatic nodule. A magnetic resonance imaging (MRI) carried out in March 2017 (Figure 1) showed a liver tumor of 2.5 × 3.0 cm in liver segments V/VI, with typical features of HCC. A chest computed tomography (CT) and a bone scintigraphy were performed in the same month and did not show signs of metastatic disease. His serum alpha-fetoprotein (AFP) at that time was 31.07 ng/ml and he was completely asymptomatic.

The first treatment offered to the patient was liver transplantation. While waiting to be included in a liver transplantation list, he received a transarterial chemoembolization (TACE) with doxorubicin in July 2017. An abdominal CT was performed one month after the procedure, showing the same tumor dimensions. His inclusion in a liver transplantation list was denied by the responsible medical council because the MELD score was low and only one nodule was detected; therefore, nodule resection was suggested as treatment. He was then submitted to a liver resection in October 2017. The histological analysis showed a well-differentiated HCC with angiolymphatic invasion. Unfortunately, the specimen margins were not free of liver cancer; therefore, the surgery was considered not curative.

In March 2018, an abdominal CT showed that the patient had a 3.4 cm liver tumor with peripheral contrast enhancement. Another bone scintigraphy confirmed the lack of metastatic disease, but a chest CT scan revealed 2 lung nodules of 0.8 × 0.6 cm in the right inferior lobe and 0.54 cm in the left inferior lobe. His AFP level was 14.53 ng/ml.

**Figure 2:**
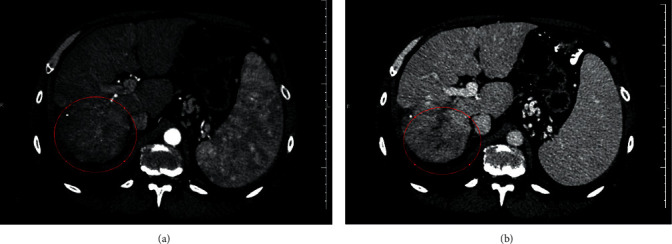
Computed tomography performed on October 1, 2018, showing the increase of the tumor dimensions. At this time, the tumor had 5.3 cm and still had arterial phase enhancement. It had led to thrombosis of the right portal vein branch. The committed segment is underscored by the red circle. (a) Arterial phase. (b) Portal phase.

Since the patient had already developed pulmonary metastasis, a systemic treatment with sorafenib was proposed. However, as we knew that the drug would be available through the public health system only after 3 or 4 months, a new TACE was performed in April 2018, when his AFP was 35 ng/ml. Sorafenib was then initiated in July 2018 (400 mg TID). An abdominal CT scan in October 2018 (Figure 2) showed that the liver tumor had increased its diameter to 5.3 cm, and a portal branch thrombosis was also detected. New metastatic nodules were found in his superior lung lobes, with 0.73 and 0.8 cm in the right and left lobes, respectively. Lymphonodal involvement was also observed, with lymph nodes of 0.63 to 1.32 cm around the aorta, 0.54 cm anterior to the vena cava, and 1.03 to 1.5 cm around the trachea and 1.46–1.55 cm hilar lymph nodes. Of note, some of the lymph nodes had central necrosis, and the AFP level was 1071 ng/ml.

Based on the good tolerance to the full-dose sorafenib treatment and the undoubtful signs of disease progression, the antineoplastic regimen was changed to regorafenib in December 2018 at the dose of four 40 mg tablets per day, 3 weeks a month. His AFP level was 1175 ng/ml.

**Figure 3:**
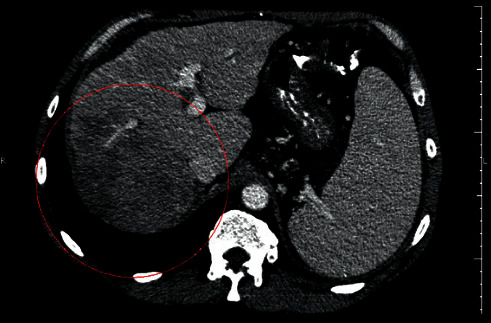
Computed tomography performed on February 18, 2019, showing a new progression of the tumor dimensions, now achieving 11.7 cm. The image was obtained during the arterial phase, but the arterial enhancement was changed to a radiolucent area underscored by the red circle.

At that time, the patient presented jaundice and complained of inappetence. His serum bilirubin level was 2.7 g/dl. The treatment was maintained and a new CT scan was performed in February 2019, showing that the liver tumor has grown to 11.7 cm, but at that time, the arterial phase enhancement was not so clear (Figure 3). A similar increase was found in the lung tumors, reaching 11.4 cm in the right superior lobe. In addition, new tumors were found at the medium lobe (2.8 cm), lingula (0.55 cm), and inferior left lobe (0.4 cm). The AFP level was 3005 ng/ml. Despite the poor initial response to regorafenib, our understanding was that the patient had not taken the medication long enough; therefore, the treatment efficacy could not be inferred. Our decision was to maintain the use of regorafenib and wait for new imaging exams.

**Figure 4:**
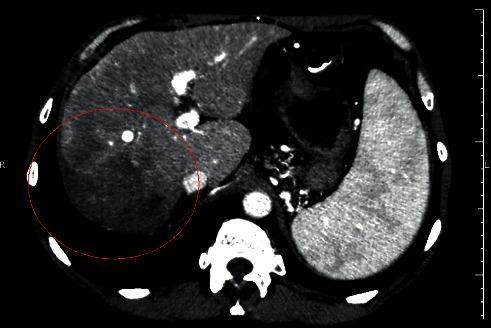
Computed tomography performed on April 8, 2019, showing a clear reduction of the liver tumor in the arterial phase, underscored by the red line. Instead of arterial phase enhancement, the tumor was now fulfilled by necrotic tissue.

In April 2019, his AFP level had dropped to 566 ng/ml. A clear reduction of the lymph nodes dimensions was documented, and the liver tumor had no more arterial enhancement (Figure 4). The lung metastases dimensions were smaller as well: the tumor in the left superior lobe had 0.55 cm, the one in the right superior lobe had 0.64 cm, the one in the medium lobe had 0.8 cm, and the one in the left inferior lobe had 0.77 cm. In January 2020, the patient is still asymptomatic and keeps all of his daily activities under regorafenib treatment.

## 3. Discussion and Conclusions

All the characteristics required to change to a second-line TKI were met by the patient described herein, who kept normal albumin levels while receiving sorafenib, so the change to regorafenib was clearly possible. Nevertheless, the increase in total bilirubin, AFP, and tumor dimensions after changing the treatment could have led to drug discontinuation. If we had stopped the treatment, this decision would have changed the patient classification to the terminal stage, in which only symptomatic measures would be prescribed, with high possibility of death within the following months. By knowing the risk of such interruption, we decided to postpone the decision to withdraw the regorafenib treatment, thus giving the patient more time until further evaluation could be performed.

When evaluating the differences between the two TKIs used by the patient, regorafenib shows a wider spectrum of kinase inhibition and is also more potent when blocking tyrosine kinases receptors, promoting a powerful blockage against angiogenic and stromal activities of the tumor [4]. Moreover, an advantage of regorafenib over other drugs proposed as second-line agents for patients with HCC is its well-known efficacy determined from preclinical and clinical studies [4, 5]. Combining these properties, a potent role against the tumor can be expected if any degree of response to sorafenib is achieved, such as the case reported above in which the arterial phase enhancement was reduced. However, regorafenib achieves high plasmatic concentrations few hours after oral administration and is accumulated throughout the treatment [4]. So, why was the tumor still growing and why did the patient develop jaundice if he had never had cirrhosis decompensation until that time?

The safety profile of regorafenib was determined in a Phase II study in which only a few patients developed hyperbilirubinemia, and it did not lead to the drug discontinuation [6]. This information made us believe that the patient would overcome the jaundice after a while, and it really happened without further complications. The concomitant increase in AFP levels leads to the hypothesis that these alterations were caused by an increase in tumor necrosis, but this assumption could not be supported by the analysis of tumor dimensions alone. A more careful observation of the images showed a progressive fading of the tumor arterial phase enhancement from Figures 1to 4. Its dimensions were reduced by the surgery and the TACE procedures, but the difference between contrast enhancement and its reduction in Figures 2 and 3 leads us to believe that the patient could still respond to regorafenib. In other words, even though the images showed an increase in nodules sizes, changes in tumor angiogenesis were also detectable, encouraging us to continue treatment with a more powerful TKI.

The aim of this case report is to show that in some cases we have to think twice before stopping a systemic treatment with TKIs such as sorafenib and regorafenib. Despite these drugs being well studied, therapy can lead to confounding findings, especially in the first weeks of treatment. In these cases, reassessing the case by a multidisciplinary team can be the key to find out that instead of a complication the patient is in fact showing signs that a good response can be expected after a while. Details observed in prior studies, lab tests, and tumor images are vital to distinguish between a total lack of response and a nonserious adverse event that should not be a reason to discontinue the treatment.

The question regarding the times to repeat imaging studies through the course of treatment is beyond the scope of this case report. Interrupting treatment or changing to a new drug are serious decisions with major impact on outcomes, especially when the patient is showing signs of a good response even when detecting these signals requires a deeper evaluation of the results. For now, the options after regorafenib are scarce, making the interruption of this drug even more problematic. Furthermore, combining locoregional and systemic therapies is possible for some strict indications but only sorafenib has been used with other therapies such as TACE, with conflicting results [7–11], thus not allowing the anticipation of results of new combinations [12].

Results obtained from clinical trials assessing sorafenib treatment suggest that the development of some adverse events may be related to time of progression and overall survival of HCC patients [13]. Changes in these outcomes can be expected after the development of skin reactions, hypertension, and diarrhea. These relations are still deeply debated and some of the proposed explanations for those are the blockage of some receptors in the affected tissues and the possible variations in the drug pharmacokinetics [13]. Until now, no similar studies are available on the influence of regorafenib adverse effects on patient prognosis. Moreover, jaundice seems to occur only during regorafenib treatment, making its pathophysiology more difficult to understand.

The case presented in this paper was submitted to TACE, liver resection, a second TACE, sorafenib, and regorafenib, a sequence of treatment modalities that have not been studied up until now. However, a clinical study about regorafenib to treat tumor recurrence after liver transplantation revealed good results, showing that new combinations of the current therapies may be an option in different situations [14]. Further clinical trials are still needed to investigate the outcomes of changing the drug schedule or reducing the daily doses and strategies used in similar cases when serious adverse events develop. Patients with no response to regorafenib therapy are still a challenge, and new drugs have been only evaluated in clinical and preclinical studies with scarce clinical data.

## Data Availability

The datasets generated and/or analyzed during the current study are not publicly available due to the hospital policy to protect the patients' data but are available from the corresponding author upon reasonable request.

## Abbreviations

AFP: Alpha-fetoprotein
CT: Computed tomography
MRI: Magnetic resonance imaging
TACE: Transarterial chemoembolization
TKI: Tyrosine kinase inhibitors.
